# Retraction of “SLCO4A1-AS1 mediates pancreatic cancer development via miR-4673/KIF21B axis”

**DOI:** 10.1515/med-2023-0890

**Published:** 2023-12-31

**Authors:** Jianxin Zhang, Yanbing Shen, Dandan Ma, Zhonghu Li, Zhiyong Zhang, Weidong Jin

**Affiliations:** Department of General Surgery, General Hospital of Central Theater Command, Wuchang District, Wuhan 430070, Hubei, China; Department of General Surgery, General Hospital of Central Theater Command, 627 Wuluo Road, Wuchang District, Wuhan 430070, Hubei, China

Retraction of: Zhang J, Shen Y, Ma D, Li Z, Zhang Z, Jin W. SLCO4A1-AS1 mediates pancreatic cancer development via miR-4673/KIF21B axis. Open Medicine 2022;17(1):253–265, DOI: 10.1515/med-2022-0418.

This article has been retracted by the publisher due to irregularities in the figures and lack of clarity regarding the origin of the images. As a result, the editors cannot guarantee the legitimacy of the presented data.

